# Evaluating the impact of employees’ perception of identity threat on knowledge sharing behavior during COVID-19: The mediation and moderation effect of social capital and reward system

**DOI:** 10.3389/fpsyg.2022.936304

**Published:** 2022-12-01

**Authors:** Michael Yao-Ping Peng

**Affiliations:** ^1^School of Economics and Trade, Fujian Jiangxia University, Foshan, China; ^2^Stamford International University, Bangkok, Thailand

**Keywords:** identity threat, knowledge sharing, social capital, reward system, high-tech

## Abstract

Quarantine policies introduced in the context of COVID-19 are affecting business operations and slowing down the flow rate of the overall economy. Different degrees and types of threats occur in both the living environment and the working environment during the epidemic prevention, which causes many additional uncertainties. The impact on employees is the identity threat from environment and organizations. This is different from the related research on the identification and impact of the threat before the occurrence of COVID-19. However, in the post-pandemic period, companies continue to strengthen important factors that can increase innovation and recovery, including the role of employee knowledge sharing. The organizational inequity and lack of organizational justice bring about the threat of internal identification in organizations. In order to ensure their own interests in organizations, employees may think twice when sharing knowledge. Therefore, this study explores the relationship among employees’ identity threat, social capital and knowledge sharing behavior from the perspective of organizational behavior. In this study, a sample of high-tech employees was conducted, and a total of 434 questionnaires were obtained. The research results show that employees’ perception of identity threat has a negative impact on knowledge sharing behavior and positively affects social capital; employees’ social capital positively affects their knowledge sharing behaviors; and organizational reward system moderates the relationship between identity threat and knowledge sharing behavior. Based on the comprehensive research findings, this research proposes corresponding theoretical and practical implications.

## Introduction

COVID-19 has been a widely concerned issue for a long time. In recent years, COVID-19 has greatly changed the economy, education, society and business of the human beings, followed by many new life and management modes (Lee et al., 2021; Cao et al., 2022; Zhao et al., 2022). Organizations and firms want to increase employees’ adaptability to changed environment and enhance their efforts made to organizations through the establishment of appropriate policies and schemes (Zhao et al., 2022). However, the environmental changes also cause constant increase of employees’ sensitivity to uncertainties and threats in the changed environment. In addition to threats to health from external environment, there are also threats from colleagues. In the business context affected by the COVID-19 pandemic, business opportunities and growth conditions also suffered enormous changes apart from the business competition environment (Peng et al., 2022). In particular, organizational innovation and knowledge innovation are the sources of competitive edges, which makes organizations attach more emphasis on employees’ innovation behaviors and knowledge innovation (Lee et al., 2021; Cao et al., 2022). However, few studies discuss the antecedent factors for knowledge sharing and innovation behaviors of employees in the context of COVID-19. Thus, this study aims to discuss the factors that affect employees’ knowledge innovation and knowledge sharing behavior in the context of COVID-19.

From the perspective of competitive threats, employees often experience identity-threatening events where they are questioned or invaded (Piening et al., 2020). Examples include questioning about their working competence or unfair evaluation of working performance from colleagues (McCord et al., 2018). These events will cause mental or emotional discomfort of workers, and may also challenge, question or lower individual competence, dignity or perceived self-worth, which thus generates the identity threat among employees (Piening et al., 2020). Scholars believe that identity threat is common to see in organizations (Petriglieri, 2011; Hall et al., 2018) and has unfavorable outcomes for workers such as the increase of antisocial behaviors and separation (Park and Shaw, 2013; Piening et al., 2020), the decrease of involvement in organizational activities behaviors and of obedience (Zellweger et al., 2013), and the harmfulness to dignity and performance (Trinkner et al., 2019). According to the IEO model, environmental factor plays an important role in input and output, and diversified risks and threats from external environment perceived by individuals will affect the expected outcomes and output. A majority of studies of knowledge management explore positive factors in environment, but few pay attention to the effect of identity threat. Thus, as risks brought by COVID-19 become a normal state, identity threat becomes an important topic to be discussed.

In view of the generality and importance of identity threats in organizations, many conceptual literature (Petriglieri, 2011) in recent years and empirical studies (White et al., 2018; Craig et al., 2019; Hay et al., 2021) were published successively. Petriglieri (2011) is the first scholar who proposed the identity-threatening process and the response theory model, arguing that identity threat is an evaluation outcome based on individual’s subjective cognition against objective events. Many subsequent empirical studies were also carried out by reference to other theories (Conroy et al., 2017; Piening et al., 2020). However, there are few studies examining the effect of employees’ perceived threats on knowledge innovation from the perspective of threats in workplace (Eisenberg and Mattarelli, 2017), so the discussion on how perceived threats lower employees’ innovation and knowledge sharing behaviors will fill in the gap of the social exchange theory. Therefore, this study seeks to discuss the effect of employees’ identity threat on knowledge innovation and knowledge sharing.

Knowledge sharing behavior is not only influenced by personal motivation, but also by knowledge sharing opportunities. Knowledge sharing behavior refers to the knowledge transfer across individuals and organizations, storage in and retrieval from knowledge base, and the process of behaviors becoming the routine and practices of organizations (Hassan et al., 2016). In addition to knowledge sharing, Lailin and Gang (2016) pointed out that knowledge transfer can promote the continuous updating and value-added of employees’ knowledge structure. Employees’ knowledge transfer is not only a process of knowledge transfer and exchange, but also a complete process of knowledge selection, assimilation, integration and application (Zhao et al., 2021). If there is no strong connection between the two, there will be a disjointing phenomenon of “the superior has policy, the inferior has countermeasure.” This phenomenon is more obvious especially in the Chinese society that attaches great importance to interpersonal relationships. Interpersonal relationships can promote formal and informal interaction among employees, exchange subject knowledge and effective resources, bring effective changes to personal knowledge development, and accumulate employees’ social capital in organizations (Ho and Peng, 2016; Chen et al., 2020). Empirical research by Hu and Randel (2014) shows that social capital is an important factor affecting knowledge sharing behavior of members; Hau et al. (2013) studied the structure of social capital and found that social capital at the structural, relational or cognitive level has a positive effect on individuals’ knowledge sharing behavior. However, this study focuses on whether the social capital can improve employees’ knowledge sharing and innovation behaviors through lowering the impact of identity threats. Therefore, this study believes that exploring the mediating effect of employees’ social capital should be of considerable importance and research value to clarify the relationship among identity threat, knowledge transfer and knowledge sharing.

As indicated in many studies of social psychology, there are several influence factors among intention and behavioral performance, leading to inconsistent outcomes caused by prediction of behavioral performance based on intention. There is also a research gap in this topic in the management field. Shneor and Munim (2019) suggested that moderating factors can be discussed based on empirical situations in behavioral studies. Although interference variables for innovation behaviors at the individual level were proposed in previous studies, only interference mechanisms related to “leadership” were discussed (Lei et al., 2019). By reference to literature in the service management field, this study puts forward an moderating mechanism, i.e., incentive system (Wei and Atuahene-Gima, 2009; Park and Yang, 2019). This study attempts to clarify whether the design of incentive systems can lead to different knowledge sharing behaviors in the job context.

According to above arguments, this study makes the following research contributions: (1) applying the social exchange theory to discuss perceived threats and risks generated by COVID-19, and discussing the effect of identity threat; (2) verifying the development process of social capital and knowledge sharing when employees perceive external threats and risks; (3) Introducing the concept of positive reward system from social exchange theory to discuss the moderating effect of reward system among identity threat and knowledge sharing.

## Literature review

### Process and response theory models for identity threat

Many scholars claim that self-identity includes personal attributes and social identities (Amiot et al., 2007). Personal attributes refer to dimensions related to sense of self, such as individual talent or capabilities; social identities refer to dimensions related to social groups where individuals belong to, such as gender or race. Since self-identity reflects self-worth, significance and evaluation, people will make efforts to maintain the positive self-identity (Jones and Volpe, 2011). When individuals perceive challenges from negative events, or raise questions about their positive self-concept (e.g., capabilities or self-worth), they will perceive that their representations or manifestations are restricted or hindered. In this case, individuals will experience self-identity threat (Yang and Konrad, 2011; Walker et al., 2017). Petriglieri (2011) described the action process of identity threat, but did not have a specific description of associations between two cognitive evaluation stages (i.e., primary evaluation and secondary evaluation) of individuals, especially how individuals affect individual outcomes (e.g., manifestation of job behaviors that are favorable to organizations) through secondary evaluation after the primary evaluation is completed. This requires further empirical studies (Brown and Coupland, 2015). As stated by the social exchange theory, exchange activities between organizations and employees will be affected by multiple external environmental factors, but identity threats may change norms, beliefs and sense of identity formed during interaction and further weakens employees’ exchange behaviors (Lin et al., 2020; Luo et al., 2021; Su et al., 2021). Prior studies mostly discussed employees’ innovation and knowledge sharing behaviors using the social exchange theory, and emphasized the effect of a variety of organizational and individual factors (Lin et al., 2020; Luo et al., 2021). But few studies have examined the identity threats of environmental factors (Su et al., 2021). Thus, this study intends to further fill in the gap in evaluation connotation of the identity threat process based on Petriglieri’s theoretical views. Besides, this study contends that employees facing identity-threatening events will not only conduct the primary evaluation for identity threats, but also conduct secondary evaluation according to the current environment so as to check whether the environment offers security assurance (Horton et al., 2014), enables them to constantly show self-identity, and uses self-identity as a basis for subsequent job behaviors. Combined with Petriglieri’s theoretical views and social exchange theory, we further discussed the correlation of identity threats with employees’ knowledge sharing behaviors.

Petriglieri (2011) argued that it is easy to produce identity threats if negative events experienced by individuals feature identity importance or experience recurrence (Brown and Coupland, 2015). Identity importance means that these negative events are closely associated with self-concept, and also generally involves individuals’ capabilities or self-worth. Examples include colleagues’ depreciation on capabilities or judgement on decisions. Since individuals often pay close attention to aspects related to self, they tend to highly perceive these negative events (Conroy et al., 2017). Experience recurrence refers to the repetition or constant occurrence of these negative events. Employees will perceive a high level of identity threats if they experience repeated challenges or doubts on their experience. Identity threats derive from employees’ subjective evaluation of negative events in workplaces (Petriglieri, 2011; Horton et al., 2014). Individuals are experiencing identity threat when individuals recognize that external negative events are likely to play down self-worth, capabilities or dignity, or dim the significance of self-identity and affect the development and continuation of self-identity (Brown and Coupland, 2015; Spyridonidis et al., 2015).

### Knowledge sharing

In addition, knowledge sharing is one of the most important behaviors in knowledge-related behaviors. As a necessary link for the transformation of individual knowledge into organizational knowledge, it can effectively enhance the ability to absorb and innovate. It is also a prerequisite for knowledge creation, and plays a key and indispensable role in the application and integration of organizational knowledge (Foss et al., 2010; Foss and Pedersen, 2019; Ritala and Stefan, 2021). Hansen (1999) defines knowledge sharing as the acceptance and provision of work-related information, expertise, experience or advice by members. From the perspective of motivation orientation, the motivation to help others is related to knowledge sharing behavior, while extrinsic and intrinsic motivation have a significant impact on knowledge sharing intention (Olaisen and Revang, 2017; Foss and Pedersen, 2019; Ganguly et al., 2019; Yong et al., 2020). Among them, motivation can enhance members’ perceived benefits of knowledge contribution, which in turn drives knowledge sharing. In addition, not only knowledge sharing motivation will affect the sharing of knowledge and experience, but knowledge sharing opportunities will also enhance the occurrence of sharing behavior (Foss and Pedersen, 2019; Ganguly et al., 2019; Zhao et al., 2021). Lilleoere and Hansen (2011) mentioned that members’ perception of sharing opportunities will affect knowledge sharing within the organization. When sharers generate knowledge sharing opportunities based on social relationships, the possible risks and costs of sharing knowledge will be reduced, thereby promoting knowledge sharing behavior (Anwar et al., 2019). Especially when individuals feel that they have convenient sharing channels, it will increase their willingness to share knowledge (Gagné et al., 2019).

There are two opposite views on knowledge sharing in previous studies. Al-Kurdi et al. (2020) believed that knowledge is the source of power, so knowledge sharing may weaken the individual’s dominant position in organizations. Pereira and Mohiya (2021) argued that knowledge sharing may cause the loss of competitive edges. On the contrary, Muhammed and Zaim (2020) pointed out that individuals who share knowledge can acquire more knowledge from the feedback from and discussion with knowledge receivers. Park and Kim (2018) contended that knowledge sharing is not only a process of learning, but also a process of helping others improve capabilities. Individuals who master knowledge caught in a dilemma about knowledge sharing because the two opposite outcomes. From the individual perspective, employees considered knowledge sharing as a threat, and are willing to share their knowledge only if there are equivalent interests for exchange (Park and Kim, 2018). However, employees will reduce or stop knowledge sharing when they recognize that knowledge sharing may cause the loss of competitive edges or may lead to unfairness (Pradhan et al., 2019). Thus, this study proposes a hypothesis as follows:

*H1*: Identity threat has a negative impact on knowledge sharing behavior.

### Social capital

Previous studies considered knowledge governance to be an important antecedent factor in the process of knowledge integration (Foss, 2007), but ignored the social relationship aspect in the process of knowledge integration. The research of Gooderham et al. (2011) and Pemsel et al. (2016) began to focus on the social relationship level of knowledge governance (for example, the social capital among members), and found that informal governance is more effective than formal governance. Ganguly et al. (2019) stated that social capital is the sum of potential and actual resources embedded in, derived from, or available from relational networks. Edinger and Edinger (2018) further interpret social capital in a team as all the resources that can be provided to the team through members’ relationships in the team’s social structure and under the broader social structure of formal and informal organizations (Alghababsheh and Gallear, 2020; Zhou et al., 2021). Therefore, the social capital of the team must be examined in a broader organizational context, and a team will have more social capital “liquidity” because of the status of its members in the entire social structure, which means that when the team needs certain resources, members can quickly and effectively use their social connections to deliver certain resources (Alghababsheh and Gallear, 2020). Most of the studies measure social capital from the relational dimension (Zaheer and Bell, 2005; Cai et al., 2011) and structural dimension (Dhanaraj et al., 2004; Moran, 2005; Pinho and Prange, 2016; De Luca et al., 2018; Chowdhury et al., 2020). In order to understand the influences of the network structures in SMEs on their overseas market performances, this study argues that as one dimension can not fully depict the profile of information and knowledge acquired from networks by SMEs (Gilsing and Duysters, 2008), so network range and network strength should be used as dimensions to present the completeness of information and knowledge benefits provided by network structure.

As argued by Petriglieri (2011), individuals are experiencing identity threats when they conclude through the primary evaluation that negative events are of high self-relevance and may cause experience reoccurrence (Brown and Coupland, 2015). With such a state of mind, individuals can perceive that their capabilities, dignity and self-worth are questioned and challenged. Prior studies also concluded that individuals have a strong driving force to protect self-concept (Amiot et al., 2007; Jones and Volpe, 2011). In this case, individuals will carry out secondary evaluation to decide measures that can reduce the harm brought by threats. As shown in this study, social capital, as the specific connotation of secondary evaluation, will facilitate individuals to seek for interpersonal relationship that can reduce threats and provide sense of safety from their social network before making decisions on subsequent behaviors (Chiu et al., 2015), because of their experience on identity threats deriving from external environment (Jones and Volpe, 2011). Thus, this study proposes a hypothesis as follows: As a result, individuals who perceive identity threats from external negative events may have a subjective evaluation that their job environment are harmful because they have found differences between external ego (negative self-concept perception) and internal ego (positive self-concept recognition); for example, individuals may perceive the unkind comments from the external environment or doubts on their capabilities and self-worth (Vardaman et al., 2018). They become sensitive to environmental cues, and are willing to truly express themselves during interaction in social relationships, which can reduce the sense of mental threats in workplaces (Tong et al., 2020). As such, employees who perceive identity threats tend to acquire more social resources and supports from social capitals so as to enhance confidence in and solutions for identifying and dealing with threats.

*H2*: Identity threat has a positive effect on employees’ social capital.

Many empirical studies have focused on the relationship between social capital and knowledge sharing (Hau et al., 2013; Hu and Randel, 2014) and found that social capital is the driving force of knowledge sharing (Liu and Meyer, 2020). From the perspective of social capital, the structure of social capital means the strength of the relationship or the frequency of interaction between an individual and other members (Sheng and Hartmann, 2019). When members have higher social capital, there will be more frequent interactions, creating sharing opportunities for members to directly contact and exchange explicit knowledge, as well as the possibility of understanding implicit knowledge (Foss and Pedersen, 2019). Therefore, the structure of social interaction can promote individuals to carry out knowledge sharing in a cost-effective way, and improve the intensity, breadth and frequency of knowledge sharing (Chiu et al., 2006; Chowdhury et al., 2020). Social capital relationships represent the quality of connection between individuals, including the degree of mutual trust or cooperation in the relationship (Bolino et al., 2002; Lin and Huang, 2022). Among them, trust based on the norm of reciprocity is the most critical factor (Le and Lei, 2018; Ganguly et al., 2019). When members have a high degree of trust, they are not only more willing to share knowledge with others, but also contain more implicit knowledge and private information (Ferraris et al., 2018; Moysidou and Hausberg, 2020). Therefore, on the one hand, based on high-quality relationships between individuals, social capital relationships are expected to encourage mutual sharing of implicit knowledge (Janowicz-Panjaitan and Noorderhaven, 2009); On the other hand, with the increase of trust, norms and cooperation in social capital relationships, it can also increase the communication opportunities of individuals and promote the sharing of explicit knowledge. Thus, this study proposes a hypothesis as follows:

*H3*: Social capital has positive effect on knowledge sharing behavior.

### Reward system

In general, incentive systems are an important tool for organizations to create psychological resources for employees (Allen and Griffeth, 2001; Chen et al., 2015). Incentive systems are job rewards given by organizations to individuals based on their performance besides salaries. Organizations also motivate employees to outdo themselves and achieve more excellent performance through incentives surpass (Malik et al., 2015; Plassmann and Weber, 2015). In the fields of knowledge sharing and organizational behaviors, rewards include financial rewards (or material rewards), and non-financial rewards. Examples of financial rewards are generally cash, stocks and other material objects, while non-financial rewards are generally symbolic rewards such as granting of titles, awarding of certificates of merit, or conferring of metals (Martono et al., 2018). The design of such reward mechanisms will affect the feeling of employees about incentive systems. Scholars in the field of organizational psychology indicated that employees have varied expectations about job outcomes due to their different social experience, leading to different recognition in incentive systems of organizations (Ngwa et al., 2019). Favorable incentive systems represent that organizations tend to recognize employees who are willing to share knowledge. The cognition of such incentive systems will affect employees’ perception over identity threats, and facilitate the possibility of knowledge sharing.

Many scholars studying organizational behaviors hold that employees develop a perception of organizations’ incentive systems by comparing their job engagement to returns, and adjusts future job engagement on this basis (Malik et al., 2015; Martono et al., 2018). Since the perception of incentive systems is a subjective thing, employees in the same organization may have different feeling about the same incentive system, thus affecting their enthusiasm in the course of work. In other words, employees can perceive a higher degree of fairness when they are well aware of the incentive system, thus reducing internal uncertainties and identity threats in organizations, and enhancing confidence in engaging in knowledge sharing (Plassmann and Weber, 2015; Rohim and Budhiasa, 2019). Employees with lower awareness of incentive systems believe that organizations do not value organizational citizenship behaviors of employees and lack care for employees. Thus, they have low confidence in and enthusiasm for knowledge sharing and will not be motivated to seek for opportunities to realize knowledge innovation (Mcfall et al., 2009; Ngwa et al., 2019). From another point of view, employees with lower awareness of incentive systems tend to regard their efforts in knowledge sharing as a significant sacrifice, because organizations do not give them corresponding rewards even if they devote a lot to knowledge sharing. Thus, this study proposes a hypothesis as follows:

*H4*: Reward system negatively moderates the relationship between identity threat and knowledge sharing behavior.

**Figure 1:**
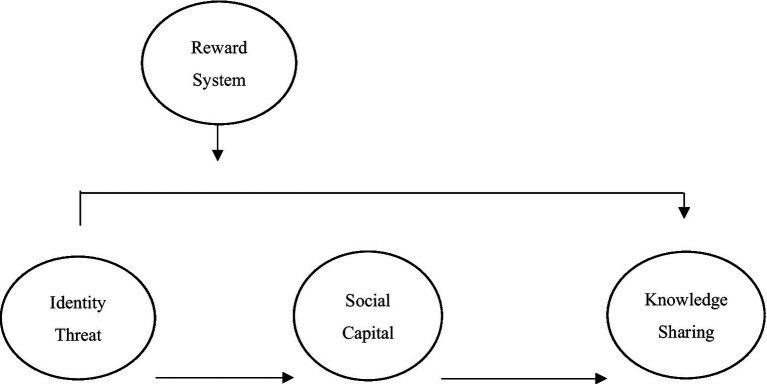
Research framework.

According to the above hypotheses, the research framework is shown in Figure 1.

## Methodology

### Sampling

This study aims to understand the impact of identity threat on employees’ knowledge sharing behavior. Different countries are responding and dealing with COVID-19 differently, so it is impracticable to take each country as a sample. This study collects samples from the Chinese mainland. There are many industries affected by COVID-19, and these affected industries may have an exogenous effect on the research result, so we only used enterprises in the high-tech industry as variables to control potential discrepancies. Thus, purposive sampling is adopted, to understand employees’ identity threat, it is necessary to focus on R&D employees, and the high-tech industry was adopted as the main sampling context. Since the purposive sampling may cause sampling bias, we set sampling conditions for the sampling strategy. First of all, we only distributed questionnaires to 20 high-tech industries in highly concentrated clusters of technology industries in the east coast of Chinese mainland. Second, we distributed different number of questionnaires to different companies, and conducted variation analysis for collected questionnaires to determine that research objects will mot have a significant impact on research variables. This study takes the R&D employees in the high-tech firms, excluding the staff in the administrative department, as the study population in order to accurately collect representative samples. In this study, copies of electronic questionnaire were sent, and 440 copies of questionnaire were collected. The 440 questionnaires were collected from high-tech firms, with each firm submitting 15–25 questionnaires. These questionnaires are sufficient in quantity for analysis during the structural equation modelling. 434 copies of valid questionnaire were obtained after excluding invalid 6. In the sample, most are male (73.1%), whose level of education is mostly master or above (68.4%), and most of them are between 30 and 40 years old (74.1%) with average working year of 6.3.

The period of sample collection was from August 2021 to October 2021. Scholars have proved that questionnaire replies at different time of period may cause data bias error. In order to verify the absence of sampling bias in this study, we test the scale using non-response bias, and compare the differences in variables of samples collected before and after September 2021. The results show that there are no differences in all variables.

Given that self-reporting measures may cause common method variances, this study evaluates common method variances through the following two types of statistical analysis. We analyzed the main composition factors using SPSS, and conducted Harman’s single factor test (Podsakoff et al., 2003). The results of factor analysis show that seven factors have characteristic values greater than 1, with the explained variation of the first factor as 35.45%, and cumulative explained variation as 67.83%. This indicates that there is no phenomenon that one factor explains most of variabilities.

### Measures

The identity threat scale is based on the identity threat developed by Aquino and Douglas et al. (2003). The measurement questionnaire contains 9 items to evaluate identity threat, such as “Judging your work in an unfair way” and “embarrassing you in front of your colleagues.” The social capital scale used in this study refers to the framework of Tsai et al. (2014), modified from the scale developed by Yilmaz and Hunt (2001) and modify the relevant vocabulary according to the educational context. The measurement questionnaire contains 11 items to evaluate the three dimensions of the social capital of the employees: “relational dimension,” “structural dimension” and “cognitive dimension,” such as “I spent a lot of time to interact with colleagues,” “I think I can count on my colleagues to do the right thing,” and “Every teacher has the same cognition on school goals.” The knowledge sharing scale is modified from the scale developed by Huang et al. (2013), and the relevant vocabulary is modified according to the educational context. The measurement questionnaire contains a total of 11 items to evaluate the three dimensions of teachers’ knowledge sharing: “knowledge sharing motivation,” “knowledge sharing opportunities,” and” knowledge sharing behavior,” such as “I am willing to share my knowledge and experience with others in a more accessible manner,” “I have many chances to build a good work team with other colleagues,” and “I often share my knowledge and experience at meetings or in discussions.” The reward system scale is modified from the scale developed by Baer et al. (2003) and Chen et al. (2015). The measurement questionnaire contains a total of 5 items to assess employees’ perception of reward system.

## Results

### Measurement

SmartPLS 3.0 and SPSS 23.0 were used to analyze the data. Before testing the hypotheses, the validity of the instrument was evaluated using convergent validity and discriminant validity. In addition, confirmatory factor analysis (CFA) was used to evaluate the measurement model. The data test results showed that the Cronbach’s α values of all the constructs were above 0.857 in Table 1. According to the study results, the reliability was significant when the Cronbach’s α coefficient exceeded 0.7, which indicated that the internal consistency of each construct was high. The AVEs and CRs of all dimensions in the Table 1 are all higher than the recommended value of 0.5 and 0.8, so all the dimensions of this study has good convergence validity. In addition, divergent validity was tested by comparing the average variance extracted (AVE) for each construct with the square of correlation coefficients. AVE for each construct was greater than the square of the related correlation coefficients, indicating the divergent validity of the constructs (Table 2).

**Table 1:** Scale measurement.

	1	2	3	4	5	6
1. Identity threat						
2. Relational dimension	−0.133					
3. Structural dimension	−0.097	0.702				
4. Cognitive dimension	−0.163	0.684	0.740			
5. Knowledge sharing behavior	−0.123	0.666	0.734	0.734		
6. Reward system	−0.092	0.608	0.687	0.661	0.687	
Mean	2.898	3.789	3.635	3.701	3.671	3.533
SD	1.141	0.937	0.980	0.955	0.971	0.987
Cronbach’s *α*	0.844	0.873	0.874	0.834	0.895	0.883
AVE	0.758	0.734	0.772	0.755	0.792	0.823
CR	0.934	0.924	0.946	0.941	0.953	0.933

**Table 2:** Indirect effect of structural model.

Paths	Std. *β*	Std. error	*t*-Value	Decision
Identity Threat → Social Capital	0.278***	0.032	4.731	Support
Identity Threat → Knowledge Sharing	−0.328**	0.046	4.200	Support
Social Capital → Knowledge Sharing	0.534***	0.063	7.832	Support
Identity Threat*Reward System→ Knowledge Sharing	−0.165*	0.022	2.132	Support

* if *p* < 0.05; ** if *p* < 0.01; *** if *p* < 0.001.

### Hypothesis testing

Before the analysis, this study first analyzed the fit of the structural model (Geisser, 1974). Then, Stone–Geisser-Criterion (*Q*^2^), coefficient of determination (*R*^2^), and standardized root mean square residuals (SRMR) is used to assess the overall model fit. In our results, *Q*^2^ values were above 0, all *R*^2^ values were more significant than 0.10, and SRMR was less than 0.08, meeting the expected criteria (Götz et al., 2010). As can be seen from analysis results of the structural model, SRMR is 0.043, and NFI is 0.933; thus, a good model fit is confirmed. Furthermore, social capital’s *R*^2^ is 0.378, and knowledge sharing’s *R*^2^ is 0.437; thus, *R*^2^ values in this study are accepted.

**Figure 2:**
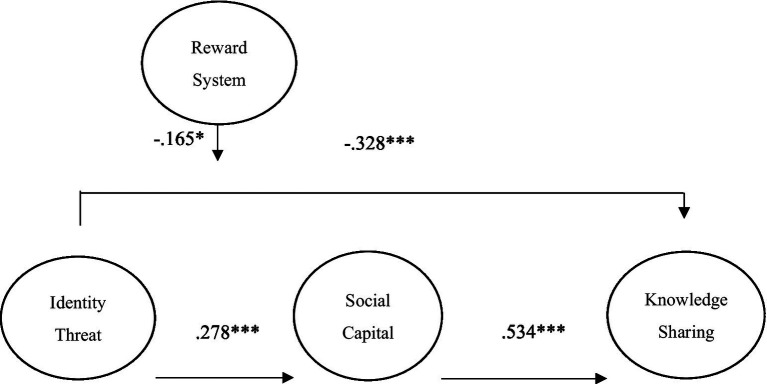
Structural model.

Figure 2 and Table 2 provide the results of testing the hypotheses. This study also tested for direct effects between the variables and derived the degree of effect. Regarding H1, the results indicate the nagetive and significant effects of identity threat (*β* = −0.328, *p* < 0.001) on employees’ knowledge sharing behavior. So H1 was supported. Moreover, the results show that employees’ perception of identity threat (*β* = 0.278, *p* < 0.001) has positive and significant effect on employees’ social capital, which supporting H2. Similarly, employees’ social capital (*β* = 0.534, *p* < 0.001) have positive impact on employees’ knowledge sharing behavior, so H3 was confirmed. Our findings also indicate that organizational reward system (*β* = −0.165, *p* < 0.05) significantly moderates the relationship between identity threat and knowledge sharing behavior; thus, H4 was supported.

**Figure 3:**
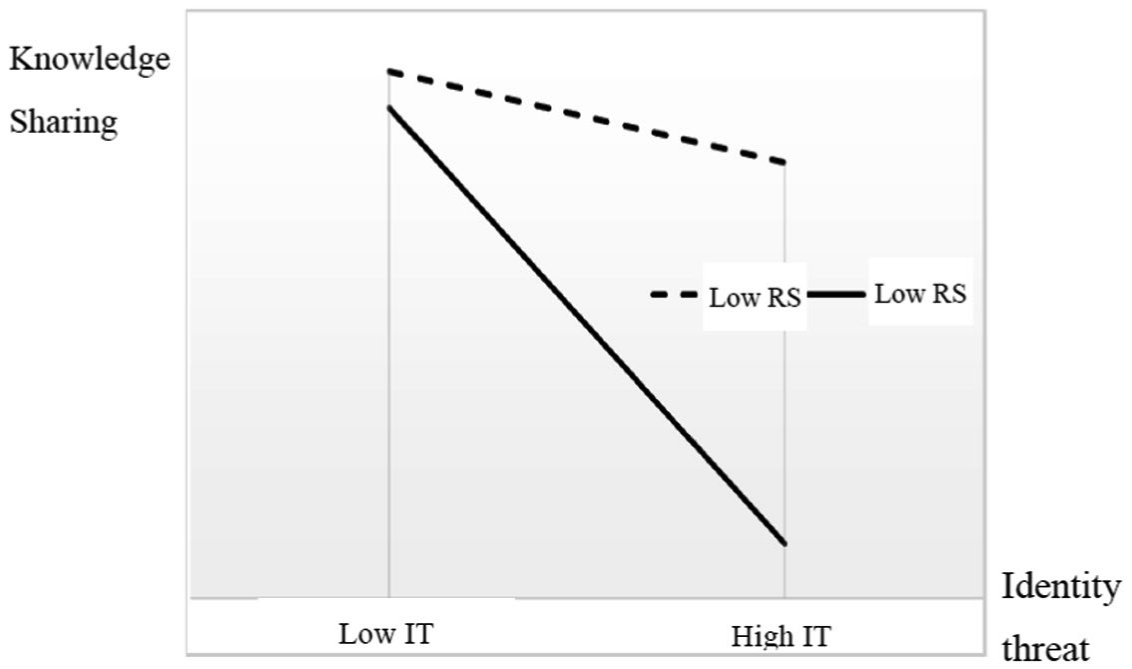
Interaction effects.

The interaction between identity threat and reward system is significant for knowledge sharing. To show the moderating effects between identity threat and knowledge sharing clearer, we plotted this significant interaction and indicated that identity threat significantly predicts employees’ knowledge sharing only when reward system is high, as shown in the simple slope chart in Figure 3.

## Conclusion

### Discussion

This study extends the views of previous identity threat scholars and argues that self-related negative work events in organizations can cause identity threat of workers (Aquino and Douglas, 2003; Douglas et al., 2003), affecting the performance of their work behavior. Researchers conduct empirical investigations into the psychological mechanisms and behavioral responses of identity threat in real organizational settings, which can supplement the research gap in the past laboratory research on identity threat (e.g., Davies et al., 2005) or qualitative research in a specific occupation (e.g., Elsbach and Kramer, 1996), such as the lack of external validity or the understanding of the dynamic process of personal adjustment (Elsbach, 2003). Based on Petriglieri’s theoretical views and social exchange theory, we propose a verifiable conceptual framework to discuss the effect of identity threats on employees’ knowledge sharing, and takes reward system and social capital as moderator and mediator, respectively. This study found that when individuals faced an identity threat event in an organization, they would experience the identity threat and evaluate the organizational situation in which they were placed, so as to examine the security degree of self-continuous presentation and determine the subsequent behavioral response. These results indicate that individuals who experience identity threat will evaluate the advantages or disadvantages of self-presentation in their situation, and then adjust their subsequent behavioral responses to avoid more threats to themselves.

This study proposes that identity threat has a negative impact on knowledge sharing behavior, but a significant positive impact on social capital. The results support the identity threat that increase the need for social capital and also reduce knowledge sharing behaviors among employees. The difference between this study and Rodionov (2021) lies that we attempt to understand whether employees will seek for more connection of external relations to reduce risks brought by identity threats when they perceive high identity threats. This study concludes that highly social support and connection can effectively threats and job insecurity arising from external environment so as to improve employees psychological stress and anxiety. This result agrees with the finding of Abbas et al. (2021). This is similar to the idea that when employees perceive a higher identity threat to the work environment and living environment, they have a distrustful attitude towards the work environment and the organization, and in order to maintain their work status and safety in the organization, they are more reluctant to provide more knowledge sharing behaviors. Ali et al. (2021) agree with this view. They indicate that employees will try to protect their knowledge and refuse to share it with other members in order to keep their competitiveness and advantages in an organization when they perceive higher external threats or job insecurity. However, when employees identify a higher degree of threat, they are more inclined to establish and maintain more diverse and close social capital. Through rich social capital, employees can obtain more information and knowledge, and provide more know-how and evaluation for the benefits and effects of knowledge sharing. Although scholars have pointed out that the higher the degree of threat identified by employees, the negative organizational citizenship behavior and the reduction of positive work behavior will be caused (Reisel et al., 2010), this study extends its research scope to positive work behaviors in organizations, and adds the mechanism of social capital to further clarify the impact of identity threat on knowledge sharing behaviors. This study can supplement Petriglieri (2011) response view of identity threat. In addition to adopting an identification-protective response to the source of the threat, or adopting an identification-reconstructing response to the threatened identification to reduce the potential harm to the identification, the results of this study found that individuals who experience identity threat may improve their judgments on identity threat through the maintenance and development of their own social capital, and then adopt appropriate behaviors to avoid the expansion of threat experience. As argued by Ali et al. (2018) and Qi and Chau (2018), employees’ willingness to knowledge sharing will depend on the socialization process within organizations, and the high sense of identity with fitting in a group will help improve communication and interaction among employees. Therefore, the process of knowledge sharing among employees depends on the role of social capital, which means that the more social capital, the more it contributes to knowledge sharing behavior among employees.

Secondly, this study found that employees’ social capital can mediate the negative effect of identity threat, indicating that the connectivity of social capital can be used to transform the impact of identity threat on personal psychology and behavior, thereby improving the generation of knowledge sharing behaviors. The results are similar to the arguments of Alghababsheh and Gallear (2020), Edinger and Edinger (2018) and Ganguly et al. (2019). They believed that employees’ social capital is not only an important source of external knowledge, but also a factor that represents the quality of relationship maintenance and interaction among employees; the intensified social capital can strengthen the cohesion between employees and organizational members and form the embeddedness effect, contributing to the inter-information flow.

Finally, this study clarified whether reward system design could buffer the negative impact of identity threat on knowledge sharing behavior. The results showed that reward system significantly adjusted the relationship between identity threat and knowledge sharing behavior, and effectively reduced the perception of organizational injustice or violation of organizational justice principles brought about by identity threat. This result is similar to the research finding of Rohim and Budhiasa (2019), who discussed positive organizational supports and culture using the social exchange theory and found that reward systems can motivate employees to improve their knowledge sharing behaviors. As advocated by scholars, an appropriate reward system will send positive signals to employees. When the rewards for knowledge sharing behaviors are given positive and positive information, employees will have inner confidence and a sense of competency, and then devote more efforts to the process of knowledge sharing.

### Implications

Due to the economic impact caused by the COVID-19 epidemic, the perceived threat of identification by employees will lead to a reduction in knowledge sharing behaviors. The relevant management implications obtained through the research structure and research results are aimed at reducing the occurrence of workplace identity threats. First of all, the organization must let all employees understand the true meaning of the identity threat, as well as the process of its generation and the impact on employees. By understanding the connotation, action process and impact results of identity threats, employees can avoid behaviors that make others feel as identity threats in the process of interpersonal interaction, such as inadvertently making unreasonable comments about colleagues or making comments about gender and racial stereotypes of colleagues, etc., thus can be used to reduce the generation of identity threats in the organization. Furthermore, the results of this study also support that the work environment in the organization is the main source of identity threats. When the identity threat is higher, there will be a high demand for the accumulation and maintenance of social capital, so that employees can give value and meaning to individual identification through social relations and social interaction. Organizations can therefore reduce employee identity threats by advocating for a positive social environment. For example, organizations can promote diverse management, friendly workplaces, or supportive leadership to reduce the occurrence of identity threats.

The knowledge possessed by the employees is the accumulation of innovation capability, and the knowledge sharing behaviors among employees are the derivative results of social capital. This study suggests that managers can effectively manage interactions and communications among employees, it will develop inter-personal relationship, and then feedback and strengthen social capital. Therefore, when the managers strengthen employees’ social capital, there will be more frequent interactions among employees, creating opportunities for employees to directly contact and exchange implicit and explicit knowledge, and the more it can promote the information circulation within the organization, enhance the effect of knowledge sharing within the organization, and form a better organizational atmosphere together.

### Limitations

Although this study provides valuable insights into knowledge sharing among high-tech employees, several limitations remain. First of all, this study discusses identity threat from the perspective of environmental uncertainty. According to different theoretical perspectives, negative factors from working environment will have more diversified variables, including job insecurity, environmental uncertainty, et. Therefore, it is suggested that future researchers can propose various key variables that are more conducive to employees’ knowledge sharing behavior based on different theories, so as to increase the richness of organizational behavior.

Social capital was regarded as an important antecedent variable in previous studies to discuss its effect on knowledge sharing behaviors. However, social capital can also be a moderator in the relationship between independent and dependent variables, and the social capital may be present in daily work, but not easy to be perceived by employees. Therefore, the moderating effect of social capital can be discussed in future studies to offer more analyses and insights.

Furthermore, due to the limitation of time and funds, this study could not study high-tech employees in different countries. Different countries may have large differences in employees’ knowledge sharing behavior due to social and cultural differences. Therefore, this study suggests that future researchers can use regional factors as moderators to explore the influence of different regional factors on employees’ knowledge sharing behavior.

## Data availability statement

The raw data supporting the conclusions of this article will be made available by the authors, without undue reservation.

## Ethics statement

The studies involving human participants were reviewed and approved by Academic committee of Foshan university. The patients/participants provided their written informed consent to participate in this study.

## Author contributions

The author confirms being the sole contributor of this work and has approved it for publication.

## Conflict of interest

The author declares that the research was conducted in the absence of any commercial or financial relationships that could be construed as a potential conflict of interest.

## Publisher’s note

All claims expressed in this article are solely those of the authors and do not necessarily represent those of their affiliated organizations, or those of the publisher, the editors and the reviewers. Any product that may be evaluated in this article, or claim that may be made by its manufacturer, is not guaranteed or endorsed by the publisher.
